# NanoCMSer: a consensus molecular subtype stratification tool for fresh‐frozen and paraffin‐embedded colorectal cancer samples

**DOI:** 10.1002/1878-0261.13781

**Published:** 2024-12-25

**Authors:** Arezo Torang, Simone van de Weerd, Veerle Lammers, Sander van Hooff, Inge van den Berg, Saskia van den Bergh, Miriam Koopman, Jan N. IJzermans, Jeanine M. L. Roodhart, Jan Koster, Jan Paul Medema

**Affiliations:** ^1^ Amsterdam UMC, Center for Experimental and Molecular Medicine, Cancer Center Amsterdam University of Amsterdam The Netherlands; ^2^ Oncode Institute, Amsterdam UMC University of Amsterdam The Netherlands; ^3^ Department of Pathology Radboud University Medical Centre Nijmegen The Netherlands; ^4^ Department of Surgery, Erasmus MC University Medical Center Rotterdam The Netherlands; ^5^ Department of Medical Oncology, University Medical Center Utrecht Utrecht University The Netherlands

**Keywords:** colorectal cancer, consensus molecular subtypes, machine learning, NanoString, prognosis biomarker

## Abstract

Colorectal cancer (CRC) is a significant contributor to cancer‐related mortality, emphasizing the need for advanced biomarkers to guide treatment. As part of an international consortium, we previously categorized CRCs into four consensus molecular subtypes (CMS1‐CMS4), showing promise for outcome prediction. To facilitate clinical integration of CMS classification in settings where formalin‐fixed paraffin‐embedded (FFPE) samples are routinely used, we developed NanoCMSer, a NanoString‐based CMS classifier using 55 genes. NanoCMSer achieved high accuracy rates, with 95% for fresh‐frozen samples from the MATCH cohort and 92% for FFPE samples from the CODE cohort, marking the highest reported accuracy for FFPE tissues to date. Additionally, it demonstrated 96% accuracy across a comprehensive collection of 23 RNAseq‐based datasets, compiled in this study, surpassing the performance of existing models. Classifying with only 55 genes, the CMS predictions were still biologically relevant, recognizing CMS‐specific biology upon enrichment analysis. Additionally, we observed substantial differences in recurrence‐free survival curves when comparing CMS2/3 patients in stage III versus II. Probability of recurrence after 5 years increased by 21% in CMS2 and 31% in CMS3 for patients in stage III, whereas this difference was less pronounced for CMS1 and CMS4, with 11% and 10%, respectively. We posit NanoCMSer as a robust tool for subtyping CRCs for both tumor biology and clinical practice, accessible via nanocmser r package (https://github.com/LEXORlab/NanoCMSer) and Shinyapp (https://atorang.shinyapps.io/NanoCMSer).

## Graphical abstract

Consensus molecular subtypes (CMS1‐4) have been identified to study colorectal cancer heterogeneity and serve as potential biomarkers. In this study, we developed and evaluated NanoCMSer, a NanoString‐based classifier using 55 genes, optimized for FF and FFPE to facilitate the clinical evaluation of CMS subtyping. Additionally, we investigated the prognostic value of CMSs using a comprehensive collection of publicly available datasets.

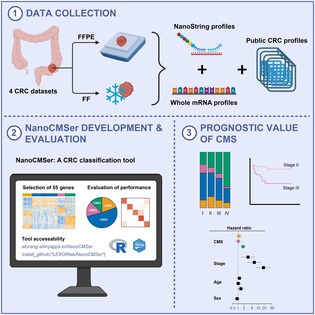

**Abbreviations**
CMS: consensus molecular subtype
CRC: colorectal cancer
FF: fresh‐frozen
FFPE: formalin‐fixed paraffin‐embedded
LogFC: logarithm of fold change
MSI: microsatellite instability
nMCC: normalized Matthews correlation coefficient
PCA: principal component analysis
RFS: recurrence‐free survival

## 1 Introduction

Colorectal cancer ranks third in cancer incidence globally, with high mortality rates [1, 2]. While pathological staging guides current adjuvant chemotherapy selection [3], it often fails to accurately predict prognosis or response. For instance, at stage III, ~ 50% of patients do not recur and do not need adjuvant therapy while ~ 25% of the patients recur despite adjuvant therapy [4, 5]. Therefore, there have been major efforts to develop additional prognostic and predictive biomarkers to improve patient selection for adjuvant therapy with the aim to reduce unnecessary toxicities and optimize treatment cost‐effectiveness [6, 7, 8].

As part of an international consortium, we proposed a transcriptomic‐based classification scheme, dividing CRCs into four biologically distinct consensus molecular subtypes (CMS1‐4) [7]. CMS1 mainly exhibits tumors with microsatellite instability (MSI) and immune cell infiltration. CMS2 and CMS3 display epithelial features, with CMS2 characterized by elevated WNT and MYC signaling, while CMS3 is marked by metabolic gene signatures. CMS4 comprises mesenchymal‐like cancers associated with stromal infiltration and poor patient outcomes [7]. This classification system was primarily built using whole transcriptomic data from fresh‐frozen samples, therefore, translating these findings into clinical practice is challenged by the lack of a suitable assay for FFPE samples with clinically relevant turn‐around times and costs.

To fully realize the potential of CMSs, a robust and reliable single‐sample classifier is needed that can process FFPE samples. This paper introduces the NanoCMSer, a NanoString‐based CMS classifier, which demonstrates high accuracy in predicting CMS in both FF and FFPE samples.

## 2 Materials and methods

### 2.1 RNA extraction

For RNA extraction from FF tissue, we followed the manufacturer's protocol using RNA‐Bee (Tel‐Test, Inc., Friendswood, TX, USA) and Trizol (ABP Biosciences, Rockville, MD, USA). Exclusion criteria for samples were set as a tumor percentage of less than 30%. When working with FFPE tissue, we prepared three 10 μm slides that were subjected to H&E staining. De‐paraffinization of tissue was carried out using Xylene, followed by macro‐dissection of the specimen to enrich for tumor tissue. RNA extraction was performed using the RNeasy FFPE kit (Qiagen, Hilden, Germany) as per the manufacturer's instructions. To assess the quality and quantity of RNA, we used the NanoDrop2000 and Tapestation (Agilent, Santa Clara, CA, USA).

### 2.2 RNAseq profiling

First, the quality of raw data was assessed using fastqc, Cambridge, United Kingdom (v.0.11.9). Trimming was performed on 12 and 11 bases for single‐end and pair‐end RNAseq, respectively. To enhance data quality, bases with a Phred score < 20 were removed using cutadapt, Uppsala, Sweden (v1.18). Reads were aligned to the reference genome (GRCh38) and expression were counted using the star, Cold Spring Harbor, NY, USA (v.2.7.4.a). The count data were log_2_‐transformed and quantile normalization was applied.

### 2.3 NanoString profiling

To quantify the expression of genes of interest, we utilized 50–150 ng of total RNA and two custom‐designed CodeSets. We included positive and negative probes to monitor the quality of runs. We performed log_2_‐transformation and quantile normalization as our standard method of normalization in this study.

### 2.4 CRC data retrieval and preprocessing

#### 2.4.1 Combined RNAseq datasets

The normalized data of RNAseq‐based publicly available datasets with CRC samples were collected, including GSE101588, GSE107422, GSE109203, GSE112941, GSE132024, GSE145429, GSE146009, GSE146889, GSE152395, GSE152430, GSE156451, GSE158559, GSE171680, GSE179042, GSE183984, GSE190609, GSE192667, GSE196576, GSE220148, GSE50760, GSE86562, GSE95132, Roelands [9] (Table S1). Only genes present in all sets were utilized to merge datasets.

#### 2.4.2 Synapse

Twelve publicly available datasets (GSE13067, GSE13294, GSE14333, GSE17536, GSE20916, GSE2109, GSE23878, GSE33113, GSE35896, GSE37892, GSE39582, TCGA [10]) and two proprietary datasets (PETACC3, KFSYSCC) which were available through Synapse (ID:syn2623706) were used to define Synapse set. We obtained normalized data from each study. Only genes present in all sets were utilized to merge datasets.

### 2.5 Gene selection for NanoCMSer

#### 2.5.1 CodeSet1

An initial selection of 134 genes was made based on differential expression analysis on TCGA dataset named CodeSet1 (Table S2). These genes were used to profile training samples from FF materials (AMC90‐FF, *n* = 41; AmsterdamUMC_CRC‐FF, *n* = 21), and when feasible, from their corresponding FFPE tissues (AMC90‐FFPE, *n* = 33; AmsterdamUMC_CRC‐FFPE, *n* = 16), utilizing the NanoString platform, which were named FF‐NanoString dataset (*n* = 62) and FFPE‐NanoString dataset (*n* = 49), respectively. Whole transcriptomic data from fresh‐frozen materials of AMC90 samples were previously generated and made publicly available (GSE33113). In this study, we also profiled the whole transcriptomic data of the AmsterdamUMC_CRC set. These datasets were used to assign CMS labels using the SSP classifier [7].

#### 2.5.2 CodeSet2

In an effort to minimize the gene count in CodeSet1 while maintaining accuracy, we utilized the feature selection capability of the glmnet package [11]. For FF materials, we first normalized quantiles of each sample in TCGA (*n* = 457) and GSE39582 (*n* = 466) datasets separately with whole FF‐NanoString dataset, using CodeSet1 genes. Next, we merged the FF‐NanoString dataset with normalized TCGA GSE39582 (*n* = 466) samples. Subsequently, 80% of FF‐NanoString dataset were randomly selected along with TCGA and GSE39582 for training an Elastic‐net model and test the remaining 20% to check the accuracy. This process was repeated 1000 times, removing genes with lower selection frequency. Next, the same procedure was implemented, but with this reduced list of genes, and continued until there was no drop in accuracy. This resulted in a total of 51 genes for FF‐NanoCMSer. Similarly, for FFPE‐NanoCMSer, using PETACC3, GSE39582 and FFPE‐NanoString dataset, 41 genes were chosen. These two lists formed CodeSet2 with probe design reported in Table S3. CodeSet2 was employed to profile test samples (MATCH‐FF, *n* = 165; CODE‐FFPE, *n* = 33). RNAseq data of FF tissues was generated for all CODE (*n* = 33) and MATCH samples (*n* = 113) to determine reference CMS labels using the SSP function in cmsclassifier package [7].

### 2.6 FF‐NanoCMSer construction

**Fig. 1:**
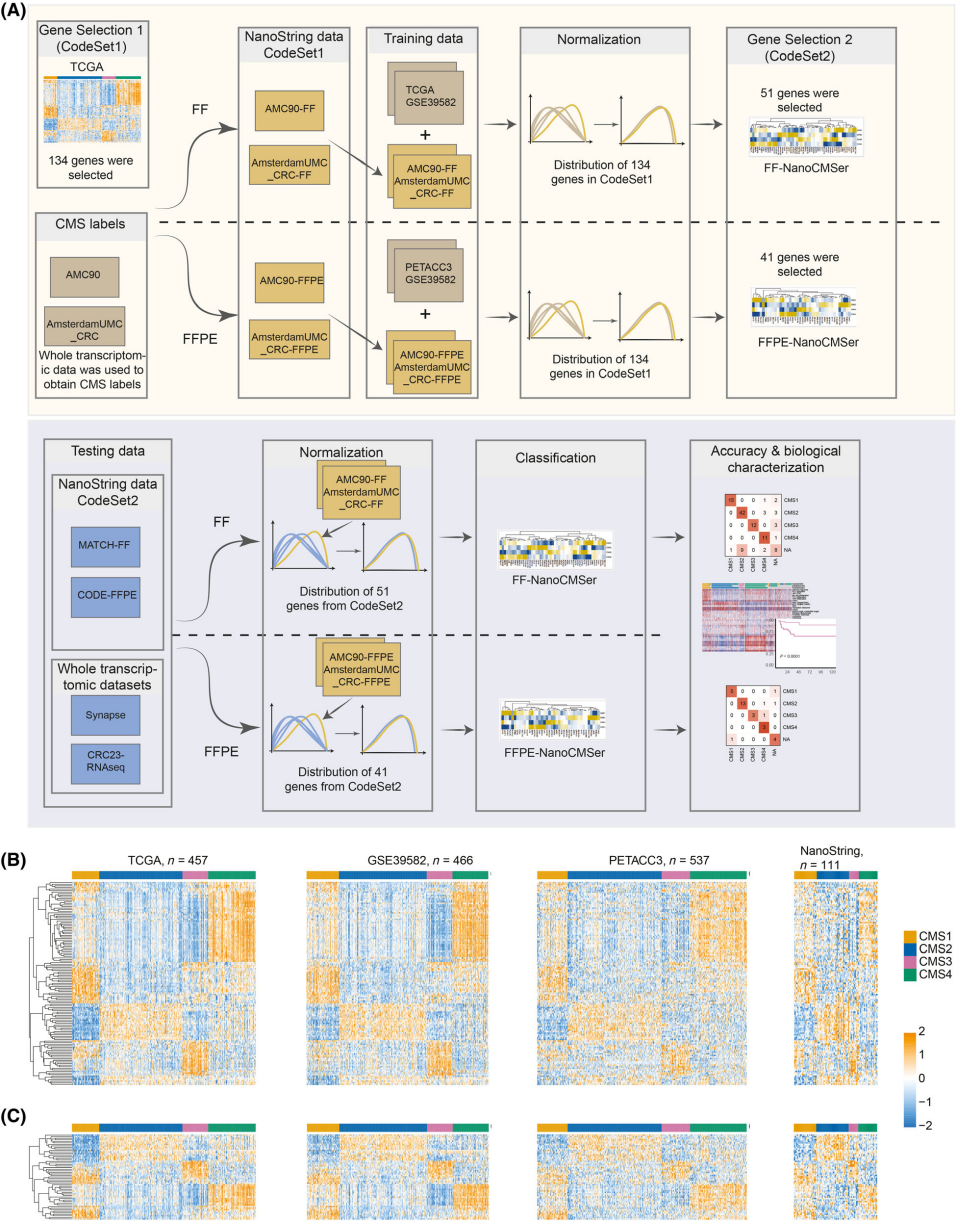
Developing the NanoString‐based CMS classifier. (A) Development of two fixation‐specific classifiers for CMS stratification, FF‐NanoCMSer and FFPE‐NanoCMSer. (B) Comparative gene expression heatmap (CodeSet1) across different platforms and fixation methods, encompassing TCGA (FF‐RNAseq), GSE39582 (FF‐microarray), PETACC3 (FFPE‐microarray), and NanoString (Mix‐NanoString) datasets. Rows represent genes, and columns represent samples. Clustering was performed on rows in the TCGA data using the linkage method and Euclidean distance, and the same gene order was applied to the other datasets for consistency. (C) Comparative gene expression heatmap (CodeSet2) across different platforms and fixation methods, encompassing TCGA (FF‐RNAseq), GSE39582 (FF‐microarray), PETACC3 (FFPE‐microarray), and NanoString (Mix‐NanoString) datasets. Rows represent genes, and columns represent samples. Clustering was performed on rows in the TCGA data using the linkage method and Euclidean distance, and the same gene order was applied to the other datasets for consistency. CMS: consensus molecular subtype; FF: fresh‐frozen; FFPE: formalin‐fixed paraffin‐embedded.

A weighted domain adaptation approach utilized FF‐NanoString, microarray and RNAseq datasets as training data. To train FF‐NanoCMSer, combined CMS‐stratified samples from TCGA and GSE39582 (*n* = 923) was used along with FF‐NanoString dataset (*n* = 62), using 51 genes in CodeSet2. Each sample of TCGA and GSE39582 was first individually quantile normalized with FF‐NanoString dataset to align with the same distribution (Fig. 1A). Next, Elastic‐net was employed using cv.glmnet function in glmnet package [11]. The Lasso coefficient for this model was optimized to 0.7, minimizing the “Misclassification Error” from a tested range of values (0.0 to 1.0 in increments of 0.1). The sample weight for NanoString was optimized to 1.5, tested against values (1.0, 1.5, 2.0, 2.5, 3.0, 4.0, 5.0, 10.0), while maintaining a constant weight of 1 for RNAseq and microarray samples.

### 2.7 FFPE‐NanoCMSer construction

For FFPE‐NanoCMSer, the CMS‐stratified PETACC3 and GSE39582 samples (*n* = 1003) were used along with FFPE‐NanoString dataset (*n* = 49), using 41 genes in CodeSet2. Quantile normalization aligned microarray samples with FFPE‐NanoString dataset, followed by training Elastic‐net model with a Lasso coefficient of 0.8 minimizing the “Misclassification Error” from a tested range of values (0.0–1.0 in increments of 0.1). Weights for NanoString were optimized to 1.5, tested against values (1.0, 1.5, 2.0, 2.5, 3.0, 4.0, 5.0, 10.0), while maintaining a constant weight of 1 for microarray samples (Fig. 1A).

### 2.8 Differential expression and gene set enrichment analysis

Gene set enrichment analysis (GSEA) was conducted using fgsea package [12]. To rank genes, we utilized LogFC (logarithm of fold change) obtained from differential expression analysis using deseq2 packages [13]. All signatures were sourced from msigdb, Cambridge, MA, USA (v7.4).

### 2.9 Single‐sample gene set enrichment analysis

We obtained pathways specific to each CMS from msigdb (v7.4) or Synapse (ID:syn2623706). Sample scores for these signatures were calculated using *Z*‐score method in gsva function (gsva package [14]). Batch effects were removed separately from Synapse and combined RNAseq datasets using sva package [15] after quantile normalization employing preprocess core package.

### 2.10 Principal component analysis

To perform principal component analysis (PCA), we utilized the prcomp function from the stats package [16]. For both Synapse and combined RNAseq dataset batch effect was removed as detailed in Section 2.9.

### 2.11 Ethics approval statement

The MATCH study was conducted with the approval of the Medical Ethical Board at Erasmus University Medical Center in Rotterdam, the Netherlands (MEC‐2007‐088). Samples were derived from patients who underwent surgical resection at Maasstad Ziekenhuis, Ikazia ziekenhuis, IJsselland ziekenhuis, Franciscus Gasthuis & Vlietland, or Albert Schweitzer ziekenhuis from 2007 until December 2017. For CODE set, samples were derived from patients who underwent surgical resection at the Reinier de Graaf Gasthuis in Delft, The Netherlands from 2007 until 2020. Ethical approval for the collection of these colorectal cancer samples was granted by the Medical Ethics Committee of Amsterdam University Medical Centers, The Netherlands (METC‐2015‐206). Written informed consent was obtained from all patients participating in this study, which was conducted in accordance with the Declaration of Helsinki and the Netherlands Code of Conduct, outlining clinical research integrity principles for institutions in the Netherlands.

## 3 Results

### 3.1 Developing the NanoString‐based CMS classifier

NanoString platform is a well‐established RNA profiling method for FFPE samples [17, 18]. To facilitate the subtyping of CRCs derived from archival clinical material, we developed a customized panel using NanoString technology.

#### 3.1.1 Experimental setup and dataset overview

We used the TCGA transcriptomic dataset (*n* = 457), to identify the initial set of genes (*n* = 134) with the potential to discriminate between CMS subtypes, referred to as CodeSet1 (Table S2) through differential expression analysis. To create a high‐performance classifier for NanoString‐based samples, we focused on generating training samples using the NanoString platform, which served both as a normalization reference (explained later) and as the primary data for training, weighted more heavily in the process. We employed CodeSet1 to profile training datasets derived from two sources: AMC90 and AmsterdamUMC_CRC, using the NanoString platform. For these two datasets, we had both FF and FFPE tissues and generated NanoString‐based datasets are referred to as AMC90‐FF, *n* = 41; AmsterdamUMC_CRC‐FF, *n* = 21; AMC90‐FFPE, *n* = 33; and AmsterdamUMC_CRC‐FFPE, *n* = 16. Additionally, we had previously obtained whole transcriptomic microarray data from fresh‐frozen materials of AMC90 samples (GSE33113). In this study, we also profiled the whole transcriptomic data of the AmsterdamUMC_CRC set. These datasets were utilized to assign CMS labels using the SSP classifier [7].

Moreover, we used the expression of genes in CodeSet1 from three whole transcriptomic datasets (TCGA, *n* = 457; GSE39582, *n* = 466; and PETACC3, *n* = 537) as co‐training samples. TCGA and GSE39582 were obtained from FF and PETACC3 from FFPE tissues. These datasets contained all four CMS subtypes (Table S4) assigned using the SSP classifier [7].

To evaluate our classifiers, we conducted testing on three independent datasets: MATCH (*n* = 113), CODE (*n* = 33), and a combined set of publicly available RNAseq profiles of CRCs (*n* = 1976). We used a final set of 55 genes referred to as CodeSet2 (Table S3) to generate NanoString‐based patient test sets. CodeSet2 is obtained from genes in CodeSet1 (Section 2.5.1). For the MATCH cohort, we utilized FF material for NanoString data generation (MATCH‐FF), while for the CODE cohort, FFPE materials were employed (CODE‐FFPE). Once again, we leveraged the whole transcriptomic data from FF tissues in both cohorts to assign CMSs using SSP classifier (Fig. 1A).

#### 3.1.2 Development of fixation‐specific classifiers for CMS stratification

To create fixation‐specific classifiers capable of accurately classifying FF or FFPE materials, we developed two NanoString‐based classifiers, denoted as FF‐NanoCMSer and FFPE‐NanoCMSer, respectively. We employed the domain‐adaptation approach to use existing data for training these classifiers by harmonization of existing data based on NanoString‐based data and setting larger weights for NanoString‐based data. When selecting co‐training samples, we aimed to use as many samples as possible to enhance classifier robustness, while ensuring the fixation type matched the intended application of the final model whenever possible. This strategy maximized the classifiers' performance and relevance. Moreover, since the NanoString platform is probe‐based and more comparable to microarray datasets, we included microarray samples in the co‐training sets to leverage this similarity. To ensure that the harmonization process did not alter the NanoString‐profiled datasets or compromise their utility for classifying NanoString‐based samples, we initially performed quantile normalization of each sample in the whole transcriptomic datasets separately using the NanoString dataset with CodeSet1 genes. This adjustment ensured that the distribution of samples from the whole transcriptomic datasets was comparable to that of our NanoString datasets. (Sections 2.6 and 2.7). As shown in Fig. 1A, we employed a dataset comprising CMS‐stratified TCGA and GSE39582 samples (*n* = 923), in conjunction with NanoString‐based datasets from FF tissues (AMC90‐FF and AmsterdamUMC_CRC‐FF, *n* = 62) to train the FF‐NanoCMSer. The expression distribution of the combined set was harmonized with that of the NanoString‐based datasets, and an Elastic‐net model was applied to select 51 genes for this model. This gene selection process aimed to minimize the number of genes while maintaining performance.

A similar approach was implemented for FFPE‐NanoCMSer, using CMS‐labeled PETACC3 and GSE39582 (*n* = 1003), alongside the FFPE NanoString datasets (AMC90‐FFPE and AmsterdamUMC_CRC‐FFPE, *n* = 49) and 41 genes were selected. By reducing the number of genes from 134 (CodeSet1) to 51 in FF‐NanoCMSer and 41 in FFPE‐NanoCMSer we aimed to prevent overfitting, hindering the classifiers performance on new data [19]. The selected genes for the two tissue types highly overlap, collectively defining CodeSet2 (Table S3).

As shown in Fig. 1A, classifying a new sample relies on a normalization step, which aligns log2‐transformed gene expression of each sample to distribution of a reference dataset specific to its fixation type, using quantile normalization method, without any median or mean centering. The reference dataset for FF‐NanoCMSer is the combined AMC90‐FF and AmsterdamUMC_CRC‐FF, and for FFPE‐NanoCMSer is the combined AMC90‐FFPE and AmsterdamUMC_CRC‐FFPE. This normalization is the same as that used for preparing whole transcriptomic data for co‐training. However, it is also crucial for classifying new, unseen samples. The function designed in nanocmser r package and Shiny app internally perform this normalization step. If raw count data are provided, the full normalization is applied. If the data are already log2‐transformed, only quantile normalization is performed (by setting ‘perform_log2 = FALSE’).

Figure 1B,C shows gene expression of CodeSet1 and CodeSet2 across subtypes in datasets profiled using different platforms, confirming a consistent pattern irrespective of profiling platform. Despite reduced quality in FFPE materials, the distinctive pattern remained discernible, effectively capturing CMSs even with a limited number of genes (*n* = 55), which validate the platform's utility for such applications. Additionally, we compared our selected genes (*n* = 55) with those selected in CMSclassifier (*n* = 803 in SSP and RF) and CMString [20] (*n* = 322). As shown in Fig. S1, five genes overlapped across all three models, potentially suggesting their importance in CMS stratification. Furthermore, 35 and four more genes overlapped with CMSclassifier and CMString, respectively, while 11 genes were uniquely selected by our method.

### 3.2 Analytical evaluation of NanoCMSer in NanoString datasets

#### 3.2.1 Coefficient of the classifiers

**Fig. 2:**
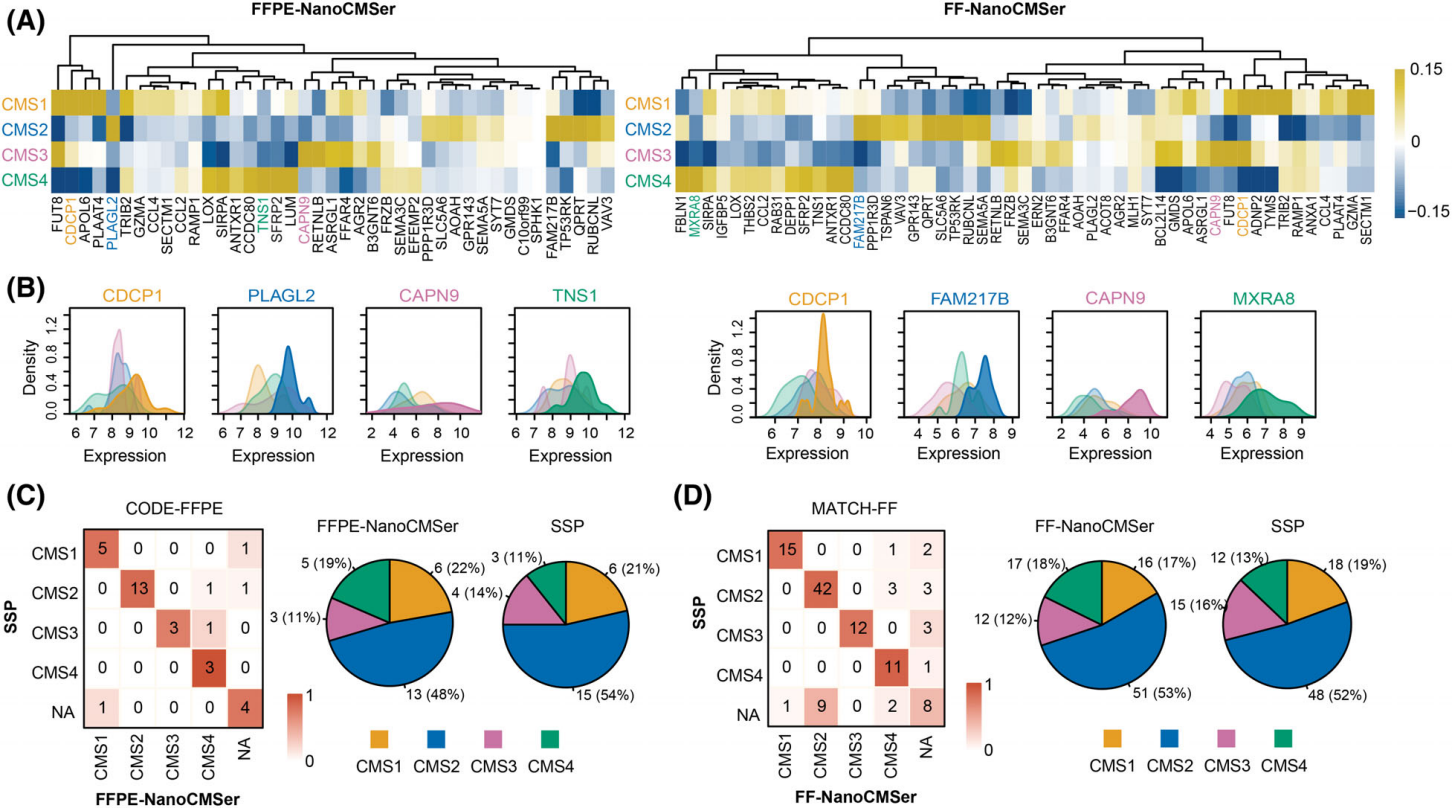
Analytical evaluation of NanoCMSer in NanoString datasets. (A) Visualization of coefficients for the final selected genes in FFPE‐NanoCMSer (left) and FF‐NanoCMSer (right) for each CMS subtype, example genes are colored according to the CMS subtype in which they have the highest coefficients. (B) Density plot illustrating the expression of example genes in the NanoCMSer models across CMS subtypes in FFPE (left) and FF data (right). (C) Concordance matrix displaying results of SSP and FFPE‐NanoCMSer stratification in CODE‐FFPE data (left) and corresponding pie chart depicting CMS distribution in each stratification model (right); (*n* = 33). (D) Concordance matrix representing results of SSP and FF‐NanoCMSer stratification in MATCH‐FFPE data (left) and corresponding pie chart showing CMS distribution in each stratification model (right); (*n* = 113). CMS: consensus molecular subtype; FF: fresh‐frozen; FFPE: formalin‐fixed paraffin‐embedded; NA: indicates not confidently classified; SSP: single‐sample predictor.

To understand the functionality of individual genes within the classifiers, we present the gene coefficients for models in Fig. 2A. Within each subtype, specific genes exhibit pronounced positive and negative coefficients, underscoring their importance in subtype discrimination. Intriguingly, certain genes contribute to discrimination of more than one subtype. For instance, FUT8 exhibits elevated coefficients in both CMS1 and CMS3, while displaying negative coefficients in others, aiding in distinction of these pairs. Furthermore, Fig. 2B shows that large positive coefficients signifying shifts in gene expression within specific subtypes, indicating higher expressions in samples of that subtype. This suggests that these coefficients may serve as subtype‐specific markers.

#### 3.2.2 Concordance of NanoCMSer and SSP

We evaluated NanoCMSer's performance using two NanoString‐based testing sets, comprising 113 FF (MATCH‐FF) and 33 FFPE (CODE‐FFPE) samples, profiled with CodeSet2. Reference CMS labels were derived from RNAseq data of matched FF tissues using the SSP classifier [7]. Comparison of NanoCMSer‐assigned labels with reference labels, for samples that were confidently labeled by both methods, revealed accuracy rates of 95% for MATCH‐FF and 92% for CODE‐FFPE, alongside comparable subtype distributions in each set (Fig. 2C,D). Due to the uneven distribution of subtypes in both sets, we calculated the normalized Matthews correlation coefficient (nMCC) [21]. The results showed a nMCC of 0.97 for MATCH‐FF (per subtype: CMS1 = 0.98; CMS2 = 0.97; CMS3 = 1.00; CMS4 = 0.92) and a nMCC of 0.94 for CODE‐FFPE (per subtype: CMS1 = 1.00; CMS2 = 0.96; CMS3 = 0.92; CMS4 = 0.87). To the best of our knowledge, these results signify the highest reported accuracy to date for CMS stratification of FFPE materials [20, 22, 23, 24]. The NanoCMSer demonstrated superior performance in accurately stratify FF/FFPE samples. Especially the latter is a crucial step towards clinical implementation as FFPE material is routinely acquired in clinical practice. Furthermore, Fig. 2C,D consistent distribution patterns between SSP and NanoCMSer predictions, indicating NanoCMSer's ability to recapitulate RNAseq‐defined reference labels.

### 3.3 Biological assessment of NanoCMSer

#### 3.3.1 Stratification of synapse

**Fig. 3:**
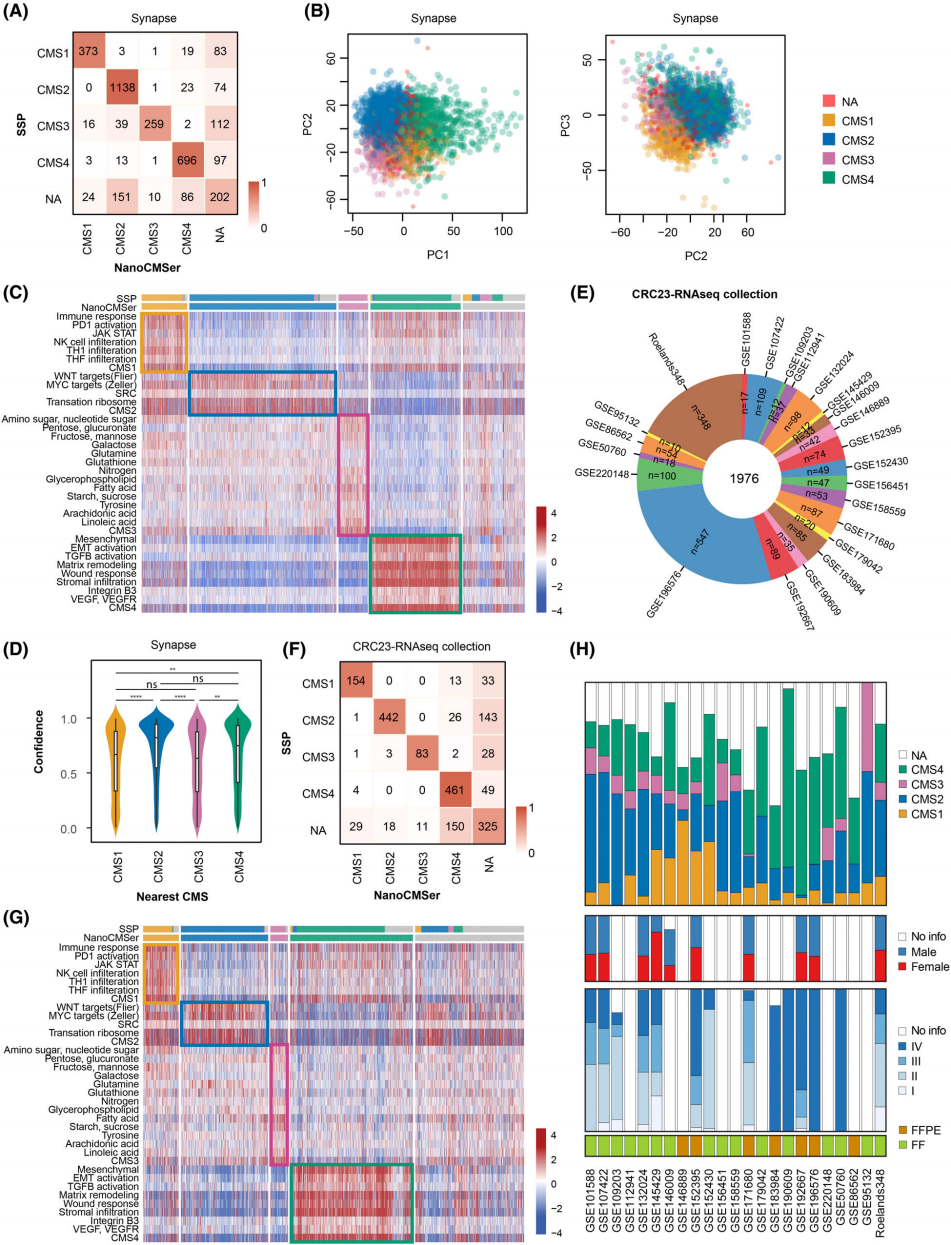
Biological assessment of NanoCMSer in combined public sets. (A) Concordance matrix comparing results of SSP and NanoCMSer stratification in Synapse data; (*n* = 3426). (B) PCA of Synapse samples, color‐coded by CMS labels. (C) Heatmap displaying ssGSEA scores of signature genes relevant to studying CMS subtypes of colorectal cancers in the Synapse dataset, ordered and segregated based on their CMS subtypes. No unsupervised clustering was performed on rows or columns. (D) Confidence scores illustrating the classifier's performance on Synapse samples. Statistical tests were conducted using the Mann–Whitney *U* test, with multiple testing corrections using the Benjamini–Hochberg method; median and interquartile range are depicted; ns: *P* > 0.05, **P < 0.01, ****P < 0.0001. (E) Overview of the CRC23‐RNAseq collection comprising 23 publicly available datasets. (F) Concordance matrix presenting results of SSP and NanoCMSer stratification in the CRC23‐RNAseq collection (*n* = 1976). (G) Heatmap displaying ssGSEA scores of signature genes relevant to studying CMS subtypes of colorectal cancers in the CRC23‐RNAseq collection, ordered and segregated based on their CMS subtypes. No unsupervised clustering was performed on rows or columns. (H) Overview of the combined dataset, including CMS labels, gender, stage, and fixation type. CMS, consensus molecular subtype; FF, fresh‐frozen; FFPE, formalin‐fixed paraffin‐embedded; NA, indicates not confidently classified; PCA, principal component analysis; SSP, single‐sample predictor.

We first stratified samples employed in identification of CMSs by Guinney et al. [7] (referred to as Synapse; Section 2.4.2), evaluating NanoCMSer. When comparing samples confidently stratified by both SSP and NanoCMSer, the accuracy rate and nMCC of NanoCMSer reached 95% and 0.97 (per subtype: CMS1 = 0.97; CMS2 = 0.97; CMS3 = 0.94; CMS4 = 0.97), respectively, as illustrated in Fig. 3A. Interestingly, a noteworthy proportion of samples eluding classification by one method were also unclassified by the other, emphasizing inherent complexity of these samples. Furthermore, we conducted principal component analysis (PCA), illustrating each sample color‐coded by its CMS subtype (Fig. 3B). This analysis demonstrated a relative separation of the CMS subtypes along the PCA components with majority of unclassified samples (shown in red) located at boundaries between CMSs.

#### 3.3.2 Comparative analysis of NanoString‐based CMS classifiers

To evaluate the performance of our newly developed classifier against previously published ones, we classified Synapse samples using NanoCMSer, CMSclia [22], CMString [20], and CRCAssigner [24]. Each of these classifiers has different thresholds for confident sample classification and varying definitions of confidence. Therefore, we standardized the proportion of predictions by using a maximum probability threshold of ≥ 0.6 for NanoCMSer, which confidently classifies 83% of samples. For the other classifiers, we selected the top 83% of samples with the highest confidence scores as defined by their respective models. As shown in Table 1, NanoCMSer, CMSclia, and CMString demonstrated similar performance levels (accuracy rate and nMCC of 92%–95% and 0.95–0.97, respectively), whereas CRCAssigner, which was initially developed to predict CRCA subtypes rather than CMS [24], exhibited the poorest performance with nMCC of 0.75. It's important to note that parts of the Synapse dataset were used to train NanoCMSer, CMString, and CMSclia, which may have influenced the reported performance. To address this potential bias, we further explore the performance of these classifiers using an independent data collection in Section 3.3.6.

**Table 1:** Comparative analysis of NanoString‐based CMS classifiers.

	CRC23‐RNAseq collection^a^				Synapse^b^			
	NanoCMSer	CMSclia	CMString	CRCAssigner	NanoCMSer	CMSclia	CMString	CRCAssigner
Accuracy rate (%)	96	91	91	57	95	92	95	60
nMCC for CMS1	0.97	0.94	0.95	0.76	0.97	0.94	0.97	0.76
nMCC for CMS2	0.97	0.94	0.94	0.76	0.97	0.97	0.97	0.74
nMCC for CMS3	0.98	0.94	0.95	0.71	0.94	0.91	0.94	0.76
nMCC for CMS4	0.96	0.91	0.91	0.75	0.97	0.95	0.97	0.81
nMCC	**0.97**	**0.93**	**0.94**	**0.73**	**0.97**	**0.95**	**0.96**	**0.75**

The bold values indicate the nMCC for all samples.

a The constant proportion of predictions (71% based on NanoCMSer) was utilized for comparative analysis.

b The constant proportion of predictions (83% based on NanoCMSer) was utilized for comparative analysis.

#### 3.3.3 Biological associations of CMSs

To further assess NanoCMSer's ability to faithfully stratify tumors based on biological characteristics [7], we scored CMS‐specific pathways in each sample. Confidently classified samples exhibited clear enrichment of the expected signatures based on the reported biological distinctions of CMSs (Fig. 3C), confirming NanoCMSer's capability in capturing relevant biological characteristics of the subtypes.

#### 3.3.4 Confidence score in NanoCMSer

As a measure of confidence in stratifications, we subtracted probability score of the second most likely subtype from the first. Scores were visualized separately for each subtype in Fig. 3D, where higher scores signify higher confidence. Although variations were observed among the subtypes showing significantly higher confidence in predicting CMS2 and CMS4 over CMS1 and CMS3, the overall confidence remained notably high, with a median value > 0.6 in all subtypes, indicating NanoCMSer capability to confidently classify all four subtypes.

#### 3.3.5 Stratification of CRC23‐RNAseq collection

To examine whether biological characteristics observed in the Synapse CRC samples, which forms the basis of CMS, persisted in independent patient sets, we compiled a comprehensive dataset comprising 23 publicly available RNAseq‐based CRC collections, named as CRC23‐RNAseq collection, collectively involving 1976 samples, consisting of both FF and FFPE samples (Table S1, Fig. 3E). We used this combined set as a new, independent collection. Since both NanoCMSer and SSP function as single‐sample classifiers, no harmonization was required between sets for classification. Therefore, we applied SSP and NanoCMSer independently to each dataset. The accuracy rate and nMCC estimation, when comparing samples labeled confidently by both methods, reached 96% and 0.97 (per subtype: CMS1 = 0.97; CMS2 = 0.97; CMS3 = 0.96; CMS4 = 0.98), respectively, as shown in Fig. 3F.

#### 3.3.6 Comparative analysis of NanoString‐based CMS classifiers using CRC23‐RNAseq collection

As discussed in Section 3.3.2, the Synapse data was used to train NanoCMSer, CMSclia, and CMString. Thus, comparing the performance of these models using the same dataset is not valid. To evaluate the performance of these models in an unbiased setting, we used the CRC23‐RNAseq collection. For consistency, we used the top 71% (based on a maximum probability score of 0.6 in NanoCMSer) of predictions in each model with the highest confidence scores. In this independent dataset, the differences in classifier performance were more pronounced, with NanoCMSer outperforming the others while maintaining similar performance to that observed with the Synapse set. This suggests the potential for overfitting in the other models, which may perform better on their training samples than on unseen testing samples. Additionally, the nMCC for CMS4 was lower in CMString and CMSclia, and to a lesser extent in NanoCMSer, compared to other CMSs. This indicates a higher rate of misclassification related to this subtype (Table 1). These findings underscore the importance of using independent datasets for performance evaluation and assessment of the generalizability of classifier models, while also highlighting the higher performance and robustness of NanoCMSer in diverse settings.

#### 3.3.7 Validation of biological associations of CMSs in CRC23‐RNAseq collection

The Synapse set characterized CMS biology, showcasing the ability to capture this biology at a single‐sample resolution in Section 3.3.2. However, there is a need for validation beyond discovery set to determine whether the identified characteristics can be replicated. Therefore, we analyzed CRC23‐RNAseq collection, assessing the presence of CMS‐specific pathways in each sample. As illustrated in Fig. 3G, confidently classified samples showed enrichment of anticipated CMS‐specific signatures. This analysis validates associations of CMSs with the reported biological characteristics.

#### 3.3.8 Diversity in the distribution of CMS across various datasets

To assess how sample characteristics effect CMS distribution, we depicted this distribution alongside gender and stage across datasets in Fig. 3H. The figure highlights substantial variations in subtype distribution associating with the characteristics. For instance, datasets with more stage IV patients tend to have higher CMS4 proportion and those with more females exhibit more CMS1 subtype. Therefore, classifiers that rely on distribution of CMS subtypes may have not been able to accurately stratify these datasets. This underscores the necessity of a classifier capable of single‐sample level operation, making its performance independent of dataset‐specific distributions.

### 3.4 Clinical and prognostic associations of CMSs in different stages

**Fig. 4:**
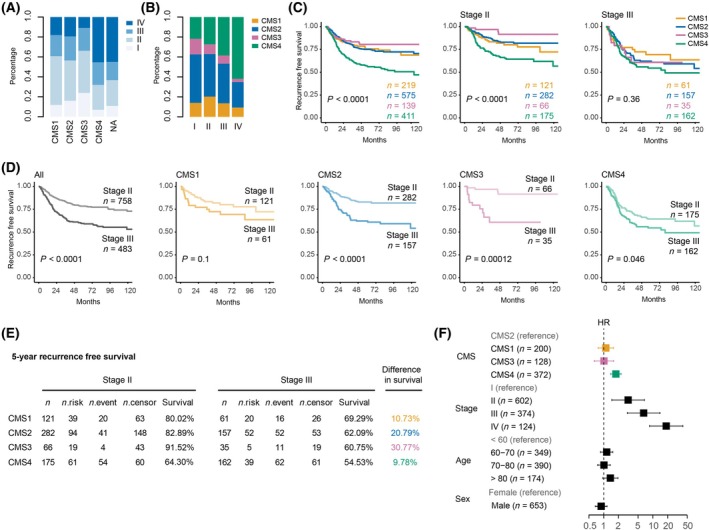
Clinical and prognostic associations of CMSs in different stages. (A) Stage distribution within each CMS subtype in the CRC23‐RNAseq collection and synapse dataset combined; (*n* = 3022). (B) CMS distribution within each stage in the CRC23‐RNAseq collection and synapse dataset combined. (C) Recurrence‐free survival values of CMS groups analyzed via Kaplan–Meier survival analysis on an aggregated cohort of the CRC23‐RNAseq collection and synapse dataset, stratified for all stages, stage II, and stage III, respectively; log‐rank test was used to examine the significance of differences. (D) Recurrence‐free survival values of each CMS subtype in an analysis between stage II and III, utilizing Kaplan–Meier survival analysis on an aggregated cohort of the CRC23‐RNAseq collection and synapse dataset; log‐rank test was used to examine the significance of differences. (E) Recurrence‐free survival analysis for stage II and stage III at 5 years per subtype. *n*, number of patient at the start of analysis; *n*.risk, number of remaining patient at the end of analysis; *n*.event, number of recurrences; *n*.censor, number of censored patients; survival: probability of staying recurrence free in 5 years duration. (F) Multivariate cox proportional hazard regression on recurrence‐free survival; bars represent the mean and the 95% confidence interval. CMS, consensus molecular subtype; HR, hazard ratio; NA, indicates not confidently classified.

Observing variation in the distribution of CMS in Section 3.3.8, we aimed to explore potential correlations with clinical attributes, specifically disease stage. Therefore, we combined clinical data from CRC23‐RNAseq collection and Synapse set (Table S1). We first displayed the percentage of patients at each stage per CMS in Fig. 4A for a total of 3022 patients. As expected, the mesenchymal subtype exhibited the highest prevalence in stage IV, signifying a propensity for advanced disease stages which is in agreement with our previous findings [25]. Similarly, unclassified patients displayed elevated stage IV prevalence, suggesting that they may associate with more advanced tumors. Conversely, CMS3 demonstrated a more pronounced presence in early stages, as depicted in Fig. 4A,B. We performed the same analyses on the Synapse and CRC23‐RNAseq collections, separately. A similar pattern was observed in both datasets, as shown in Fig. S2A–D, although the CRC23‐RNAseq collection contains a higher number of stage IV samples.

To explore whether the adverse prognosis associated with CMS4 is primarily attributed to its prevalence in advanced stages we conducted Kaplan–Meier survival analysis for the entire sample population, and separately for stage II and III (Fig. 4C). As expected, CMS4 showed significantly poorest outcome in recurrence‐free survival (RFS) irrespective of stage. Although this trend persisted in stage II, the survival curves for all CMSs converged in stage III, indicating no significant differences in their RFS.

To gain a better understanding, we presented Kaplan–Meier plots for each CMS, comparing RFS per stage in Fig 4D,E. CMS1 and CMS4 showed only a slight reduction in prognosis when transitioning from stage II to III. Specifically, Fig. 4E illustrates that the probability of recurrence after 5 years increased by 11% in CMS1 and 10% in CMS4 for patients in stage III. This increase was significantly more pronounced for epithelial subtypes, with a 21% increase in CMS2 and a substantial 31% increase in CMS3. Moreover, we tested a multivariate survival model to demonstrate the association between CMS and recurrence‐free survival (RFS), considering age, sex, and stage as other predictors. We conducted the Schoenfeld test to evaluate the proportional hazards assumption and found that sex violated this assumption (Fig. S2E). To account for potential survival differences between various CMS subtypes, while also considering other predictors and the time‐varying effect of sex, we included both a time‐independent term and a time‐dependent interaction term (modeled as a linear function of time) for sex in the Cox proportional hazards model, along with other predictors (Fig. 4F). This analysis confirmed the prognostic significance of CMS4, which was associated with the poorest outcome after adjusting for other predictors. Collectively, these observations suggest that prognostic outcomes of CMS4 are stage‐independent, whereas unfavorable prognoses for epithelial subtypes mainly emerge in advanced stages. Thus, combining stage and CMS can offer a better outcome prediction.

## 4 Discussion

In the evolving landscape of cancer treatment, personalized medicine has gained prominence. Despite this, the TNM staging system remains central to treatment decisions in non‐metastatic CRCs. The Consensus Molecular Subtyping offers a promising avenue, categorizing CRC into distinct subtypes with unique biological features. Integrating CMS with TNM staging holds potential for refining tailored treatment strategies in non‐metastatic CRC, especially given its ability to predict response and recurrence risk. Earlier studies have suggested that understanding CMS can help predict treatment response of disease recurrence, particularly in peritoneal metastasis [26, 27]. The CMS4 subtype, recognized as the mesenchymal subtype, is associated with extensive stromal invasion and poor survival rates [28]. Previous studies have investigated the therapy efficacy in CMS subtypes. It has been shown that only CMS2‐like patients benefit more from oxaliplatin [29], while CMS4 patients in stage IV appear to be more sensitive to FOLFIRI [30]. Furthermore, monotherapy with cetuximab does not benefit CMS4‐like patients, regardless of activating mutations in KRAS [31]. Our recent study also identified CMS3 patients in stage III as a group that does not benefit from adjuvant chemotherapy [32]. Therefore, CMS classification can be a valuable tool for predicting therapy response and exploring new treatment strategies.

Despite its promise, the wider implementation of CMS classification in clinical settings faces challenges due to the lack of a rapid and cost‐effective assay for classifying archival samples. Previous studies have explored NanoString‐based classifiers for archival materials, highlighting the platform's reliability. Morris et al. [22] achieved 80% accuracy rate in a NanoString‐based classifier for archival material. Ragulan et al. [24] successfully utilized the NanoString platform for CRCA classification, showcasing its potential for CRC subtyping. Similarly, Marisa et al. [23] developed a Random Forest‐based classifier using the NanoString platform, and 69% of the samples were assigned to a CMS subtype with probability ≥ 0.5. While some studies reported slightly lower accuracy in CMS subtyping, the collective evidence underscores the platform's value in advancing our understanding of CRC and guiding personalized treatment strategies.

This study introduces NanoCMSer, a NanoString‐based CMS classifier designed for both FF and FFPE tissues. Leveraging NanoString technology, our classifier demonstrates remarkable accuracy in stratifying both fixation type. Notably, it achieves the highest reported accuracy for FFPE tissues and outperforms existing classifiers, indicating its potential for clinical evaluation of CMS subtyping.

NanoCMSer's effectiveness extends to exploring the distribution of CMS subtypes across diverse datasets, emphasizing the necessity for a single‐sample classifier unaffected by dataset‐specific variations. The clinical associations reveal intriguing patterns, with mesenchymal subtypes demonstrating poor outcomes regardless of disease stage. In contrast, epithelial subtypes manifest unfavorable prognoses in advanced stages. This insight suggests that the combination of stage and CMS as biomarkers can provide a more understanding for predicting outcomes. To ensure that this newly developed classifier is accessible to the wider community, we created a publicly available r package, NanoCMSer, and a user‐friendly Shinyapp, accessible via github.com/LEXORlab/NanoCMSer and atorang.shinyapps.io/NanoCMSer, respectively.

Acknowledging limitations, survival analysis in our study was conducted without considering patient treatment due to missing information in most datasets. Further analysis is warranted to explore observed differences in outcomes per subtype, paving the way for comprehensive insights into the implications of CMS in treatment response and patient outcomes.

There are still several unmet criteria for the implementation of CMS in clinical settings. Despite efforts by us and others to mitigate the laboratory extensiveness and informatics procedures through the use of NanoString technology and a user‐friendly classification system, challenges remain. First, CMS subtyping is influenced by the tumor microenvironment, particularly evident in CMS1 (immune infiltration) and CMS4 (stroma infiltration). This variability in the tumor microenvironment makes the sampling site critical for CMS stratification. As previously demonstrated, stroma infiltration can differ between tumor specimens, leading to variability in CMS labels even within samples from the same patient [33, 34]. Additionally, across all methods, a significant proportion of patients cannot be confidently labeled into any of the CMS subtypes, which presents a critical limitation for the clinical implementation of CMS subtyping. Several factors could contribute to the unclassified status of these samples, including technical variability, quality of gene expression data, or the possibility that these samples exhibit molecular features not captured by the current CMS subtype framework or display mixed characteristics. This limitation underscores the urgent need for further refinement of the subtyping before it can be considered for implemented in clinical practice. Achieving consistent subtyping for nearly all patients is essential and superior for its utility in subtype‐based treatment decisions. These need to be addressed to warrant the clinical implementation of CMS subtypes.

## 5 Conclusions

In conclusion, NanoCMSer emerges as a valuable tool for CRC stratification, addressing some of the challenges in adopting CMS classification in routine pathology. The integration of CMS and NanoCMSer into clinical practice holds promise for refining treatment strategies and enhancing prognostic precision in the era of personalized cancer care. Future studies, considering treatment variables, will contribute to better understanding of clinical implications of CMS subtypes.

## Conflict of interest

The authors declare no conflict of interest.

## Author contributions

The conceptual framework of the study was devised by AT, SW, JK, JPM, MK, JNI, and JMLR. SW, IB, VL, and SB were responsible for the collection and profiling of patients' data. Data analyses and interpretations were conducted by AT, JPM, JK, and SH. All authors contributed to the writing and review of the manuscript, and all authors have reviewed and approved the final version.

### Peer review

The peer review history for this article is available at https://www.webofscience.com/api/gateway/wos/peer‐review/10.1002/1878‐0261.13781.

## Supporting information


**Fig. S1.** Overlapping genes in different models.

**Fig. S2.** Association of CMS and stage.

**Table S1.** Clinical information and consensus molecular subtypes of Synapse and CRC23‐RNAseq collection.

**Table S2.** Probe design for CodeSet1.

**Table S3.** Probe design for CodeSet2.

**Table S4.** Distribution of CMS subtypes across datasets within the training set.

## Data Availability

Data generated in this study are available in Gene Expression Omnibus (GEO) repository. The GEO accession numbers of NanoString‐based profiles of AMC90, AmsterdamUMC_CRC, MATCH and CODE are GSE261104, GSE260968, GSE261107, and GSE261108, respectively. The GEO accession numbers of RNAseq profiles of AmsterdamUMC_CRC, MATCH and CODE are GSE262670, GSE262671, and GSE262672, respectively. NanoCMSer is publicly available as nanocmser r package (https://github.com/LEXORlab/NanoCMSer) and Shinyapp (https://atorang.shinyapps.io/NanoCMSer). Codes are accessible via github.com/LEXORlab/NanoCMSer_Scripts.
