# Supplementary material for: NanoCMSer: a consensus molecular subtype stratification tool for fresh‐frozen and paraffin‐embedded colorectal cancer samples
Source: Mol Oncol. 2024 Dec 25;19(5):1332–46. doi: 10.1002/1878-0261.13781 (PMC12077266; doi:10.1002/1878-0261.13781)
Supplement: Supplementary file 1 — Fig. S1. Overlapping genes in different models. [file MOL2-19-1332-s002.pdf]

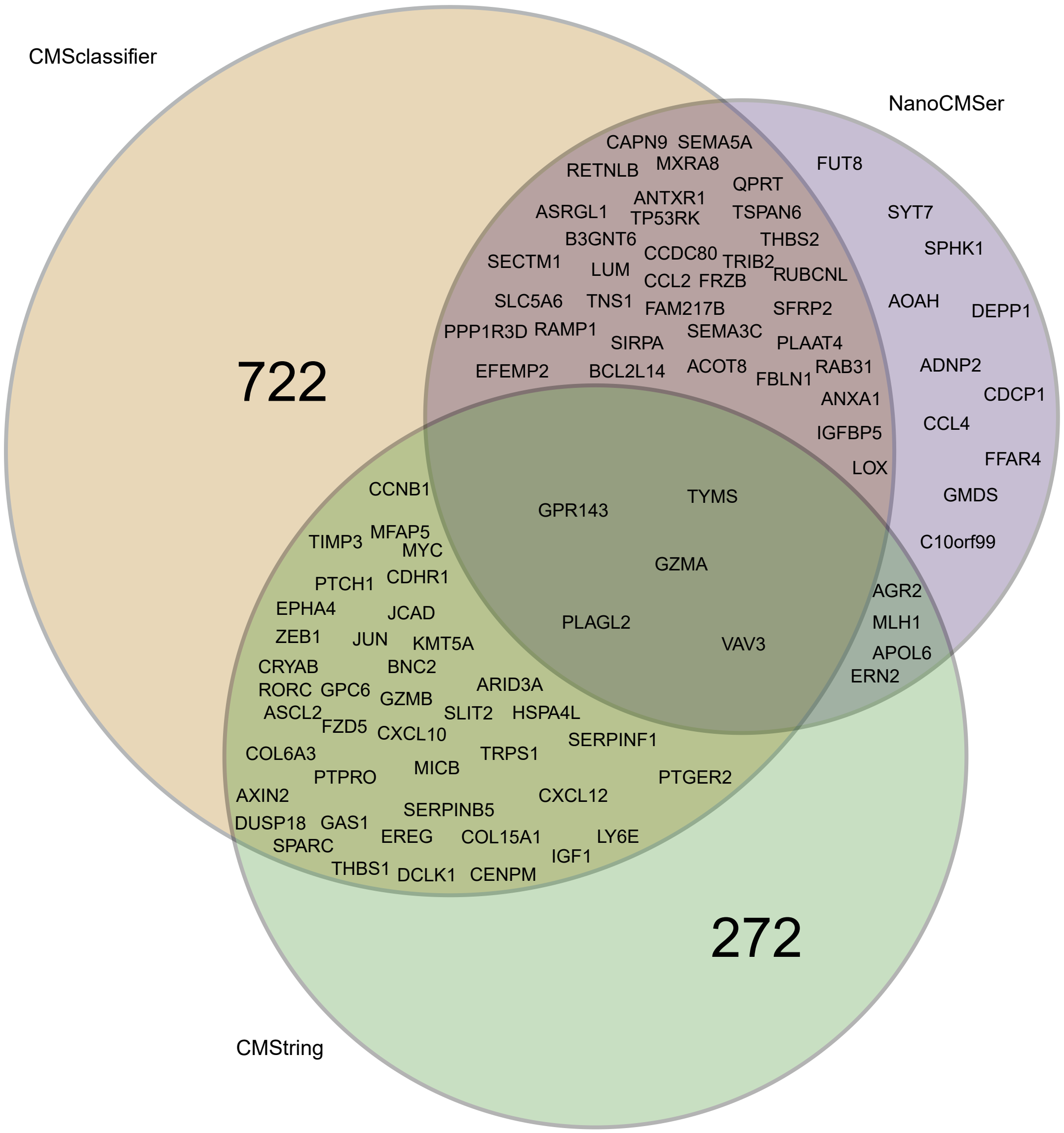


**Figure S1.** Overlapping genes in different models.

Venn diagram demonstrating overlapping genes in NanoCMSer, CMSclassifier (combination of RF and SSP), and CMSstring.

Abbreviation: RF: random forest model; SSP: single sample predictor.
