# Supplementary material for: NanoCMSer: a consensus molecular subtype stratification tool for fresh‐frozen and paraffin‐embedded colorectal cancer samples
Source: Mol Oncol. 2024 Dec 25;19(5):1332–46. doi: 10.1002/1878-0261.13781 (PMC12077266; doi:10.1002/1878-0261.13781)
Supplement: Supplementary file 2 — Fig. S2. Association of CMS and stage. [file MOL2-19-1332-s006.pdf]

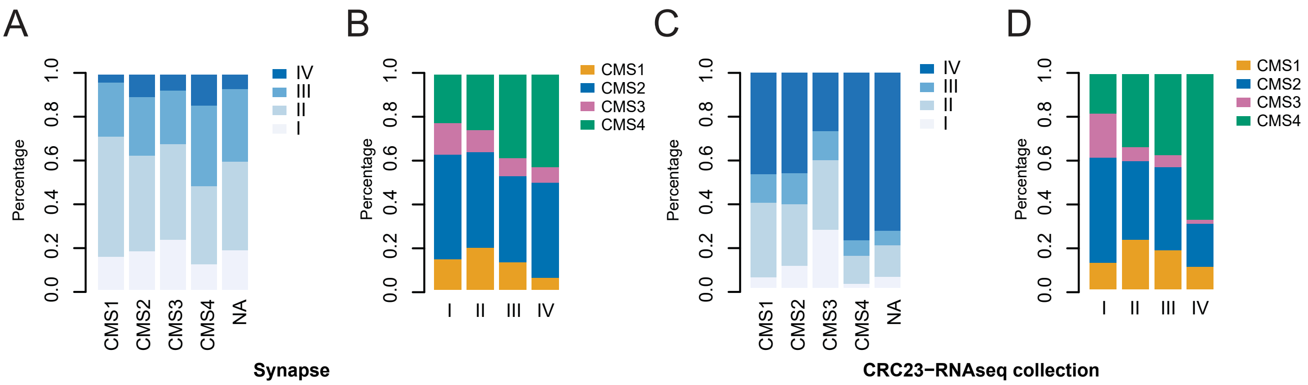

Figure S2. Assocation of CMS and stage.

A. Stage distribution within each CMS subtype in the synapse dataset
B. CMS distribution within each stage in the synapse dataset
C. Stage distribution within each CMS subtype in the CRC23-RNAseq collection
D. CMS distribution within each stage in the CRC23-RNAseq collection

Abbreviation: CMS: consensus molecular subtype; NA: NA indicates not confidently classified.
